# The public’s comfort with sharing health data with third-party commercial companies

**DOI:** 10.1057/s41599-020-00641-5

**Published:** 2020-11-11

**Authors:** M. Grace Trinidad, Jodyn Platt, Sharon L. R. Kardia

**Affiliations:** 1 Department of Learning Health Sciences, University of Michigan Medical School, Ann Arbor, MI 48109-2054, USA.; 2 University of Michigan School of Public Health, Ann Arbor, MI 48109-2054, USA.

## Abstract

Healthcare systems are using big data-driven methods to realize the vision of learning health systems and improve care quality. In so doing, many are partnering with third-party commercial companies to provide novel data processing and analysis capabilities, while also providing personal health information to a for-profit industry that may store and sell data. In this research we describe the public’s comfort with sharing health data with third-party commercial companies for patient and business purposes and how this comfort is associated with demographic factors (sex, age, race/ethnicity, education, employment, income, insurance status, and self-reported health status), perceived healthcare access, and concerns about privacy. We surveyed the US public (*n* = 1841) to assess comfort with sharing health data with third-party commercial companies for patient or business purposes and examined whether there was a difference between comfort with data sharing for patient or business purposes. Univariate and stepwise regression modeling is used here to estimate the relationship between comfort with third-party commercial companies for patient and business purposes (outcomes) and demographic factors, self-reported health status, perceived healthcare access, and privacy concerns. The public is more comfortable sharing health data with third party commercial companies for patient purposes as compared to business purposes (paired *t* = 39.84, *p* < 0.001). Higher education was associated with greater comfort with sharing health data for patient purposes (*β* = 0.205, *p* < 0.001) and decreased comfort with sharing health data for business purposes (*β* = −0.145, *p* = 0.079). An inverse relationship exists between privacy concerns and comfort with sharing health data for both patient (*β* = −0.223, *p* < 0.001) and business purposes (*β* = −0.246, *p* < 0.001). Participants ages 45–59 were less comfortable sharing health data with third party commercial companies for patient purposes (*β* = −0.154, *p* = 0.0012) than participants aged 18–29. Proactive acknowledgment of privacy concerns and better communication of the steps being taken to protect the privacy of health data can increase patient comfort. Healthcare systems may be able to increase public and patient comfort with sharing health data with third-party commercial companies by emphasizing the patient-centered benefits of these partnerships.

## Introduction

### Background.

In the fall of 2019, Google and Ascension announced a data partnership called “Nightingale.” As part of the effort, Ascension, the largest non-profit healthcare system in the United States, moved identifiable patient records onto Google’s cloud servers to begin data analysis on a subset of Ascension’s patient population of 50 million people (Copeland and Needleman, 2019). News coverage of the partnership included language such as “secretly gathering personal health records (Griggs, 2019)” and “Google: You can trust us with the medical data you didn’t know we already had (Brodkin, 2019)”. What likely began as an exciting data–discovery partnership has since devolved into a full investigation by the Office for Civil Rights in the Department of Health and Human Services (Brodkin, 2019). This response by the public, however, was not unprecedented. At the time of the announcement in November 2019, Google and the University of Chicago were being sued for the use of identifiable patient records without consent (Wakabayashi, 2019) while news coverage revealing the details of Sloan Kettering’s external startup venture known as Paige.AI resulted in an internal, system-wide review of all third-party commercial company data sharing agreements. Both incidents were linked to a breakdown in community trust (Singer and Wakabayashi, 2019; Vincent, 2019).

Despite the negative attention of these exemplars, partnerships and electronic personal health information (ePHI) data sharing agreements like these are key to realizing the potential of big data efforts in healthcare: personalized medicine, better understanding of rare diseases, and reduction of prescription errors, among other efforts. As healthcare systems expand their data and technology ventures and third-party partnerships, the ethics of these partnerships and the responsibility of the healthcare system to the patient are being questioned. Studies have highlighted the public’s concern about the use of health data by third-party commercial companies and have shown that sharing health data may lead to the deterioration of patient trust (Critchley et al., 2015), that public trust in commercial health companies is low (Castell and Evans, 2016), and that patients would consider leaving their health system for another if their current hospital shared their personal data without consent (Lewis and Bays, 2019). However, there remains little insight on what healthcare systems can do to manage or mitigate public concerns about the use of their health data.

The aim of our study is to first characterize the public’s comfort with sharing health data with third-party commercial companies, and then examine whether expressed purpose of data use—for patient purposes or for business purposes—impacts the public’s comfort with third-party commercial companies. This study contributes to the growing body of research on how the general public perceives health data partnerships with commercial companies and provides insight on how healthcare systems can improve how data sharing partnerships are managed.

Although the distinction between patient-centered uses and business uses of health data are closely linked, healthcare systems may be operating under the assumption that patients are aware of how business-centric uses of patient data likely lead to improvements in patient care. For example, in the case of Sloan Kettering, healthcare organizations may emphasize the innovative nature of the partnership and the benefits accrued to the health system instead of the tangible, concrete improvements to patient care (CooperKatz, 2018). Previous studies have dichotomized patient uses and business uses even though these two concepts are intertwined, interrogating comfort when data is shared for patient purposes, i.e., to improve care, diagnosis, or treatment, versus comfort when data is shared for business purposes, i.e., the sale of de-identified data for artificial intelligence efforts or marketing. This division allows for examination of the effect of communicated purpose of use on comfort or willingness to share health data with third-party commercial companies (Anderson and Agarwal, 2011).

Previous research on patient willingness or comfort with sharing healthcare data indicate patient reservations about the use of healthcare data outside of those services needed to provide direct care (Kim et al., 2019) and concern about the motivations behind the use of health data (Stockdale et al., 2019). Despite these reservations, however, patients are largely supportive of research efforts and generally look forward to the potential healthcare insights offered by large patient data sets (Doukas and Hardwig, 2014; Reynolds and Nelson, 2007; Shavers et al., 2001), so long as the effort is of “high value with an ‘overall impact on society’ and considers whether ‘just a few hundred [people] or several thousands’ would benefit” (Damschroder et al., 2007). Patients are also more willing to share health data even with commercial companies if the potential health benefits to the public are clear (Anderson and Agarwal, 2011; Castell and Evans, 2016), but desire more control over their health data if that data will be used for profit-generating research or for private benefit (Castell and Evans, 2016; Willison et al., 2009),

To characterize comfort with sharing health data with third-party commercial companies, we explore the impact of privacy concerns, perceived healthcare access, self-reported health status, and demographic characteristics on comfort with sharing health data with third-party commercial companies. Highly negative emotions about health status (personal experience with a past or present cancer diagnosis) is associated with an increased willingness to share PHI with pharmaceutical companies for clinical trial research (Anderson and Agarwal, 2011). However, studies on the effect of health status broadly on participant willingness to share health information have been contradictory—in one study, patients with self-rated fair or poor health were less willing to share their health information (Weitzman et al., 2010). In a study involving HIV patients, perceived healthcare access, or the patient’s satisfaction with their ability to access necessary healthcare, was associated with increased willingness to share PHI (Teixeira et al., 2011). Differences in willingness to share personal information has also been found according to educational attainment (Blank et al., 2014; Kim et al., 2019; Sheehan, 1999) and income (Lee et al., 2016; O’Neil, 2001). These studies have found that as educational attainment and income increases, willingness to share information decreases. Overall willingness to share information is modified by privacy concerns—intuitively, individuals with greater privacy concerns express greater reluctance to share data than those with less privacy concerns (Anderson and Agarwal, 2009).

In this study, we characterize the public’s comfort with sharing healthcare data with third-party companies when data sharing is expressed in terms of patient purposes, i.e., to improve care, diagnosis, or treatment, versus comfort when data is expressed in terms of business purposes, i.e., the sale of de-identified data for artificial intelligence efforts, with the goal of better understanding how presentation of third-party commercial partnerships affect comfort with sharing health data. We specifically examine how privacy concerns, perceived healthcare access, self-reported health status, and demographic factors are associated with both types of comfort with data sharing in order to provide insight on how healthcare systems and policy makers may navigate future data partnerships.

## Methods

We surveyed a sample of U.S. residents using the National Opinion Research Center’s (NORC) probability-based, nationally representative sample of US adults. NORC’s national sample frame employs a two-stage probability sample design to select a representative sample of households in the United States, over-sampling African American, Hispanic populations, as well as households 200% below the federal poverty level. Survey recruitment and deployment was done in May 2019. Data collection was completed by June 2019. Eligible participants (at least 21 years old and able to read and write in English) were contacted via email to participate in the online survey, resulting in a total of 2157 participants (66% response rate).

NORC calculated post-stratification weights according to US Census demographic benchmarks for age, sex, household income, education, as well as race and ethnicity to reduce sampling bias. For the purposes of this paper, records with missing responses to one or more of the questions used in this analysis were not included, resulting in a final analyzed sample of 1841 responses. This study protocol was approved by the University of Michigan Health Sciences Institutional Review Board.

### Survey and measurements used in this study.

Variables used in this study were derived from a 20-min, 164-item survey created to examine knowledge, attitudes, and beliefs about data sharing. Privacy measures were adapted from Anderson’s work on consumer willingness to disclose personal health data and the California Health Foundation’s 2005 National Consumer Health Privacy survey (Anderson and Agarwal, 2011; Bishop et al., 2005). Privacy measures also include questions about deception and medical mistrust (Boulware et al., 2003; LaVeist et al., 2009) and have been used in previous studies (Platt et al., 2018). Measures for perceived healthcare access and comfort with third-party commercial companies were reviewed by experts and validated using cognitive interviews.

All survey takers were shown a short (90 s) animated video describing how health data is shared through the duration of care in the context of precision oncology—with insurers, billers, and analysts learning from the outcomes of treatment. Definitions of important terms such as “healthcare system”, “healthcare providers”, “electronic health record”, “de-identified health information [or biospecimens]”, and “commercial companies” were provided to survey participants wherever those terms appeared. “Commercial companies” was defined for respondents to this survey as “third-party companies that are not part of a hospital. For example, a third-party commercial company may conduct genetic tests and analyze information for a hospital or healthcare provider for a fee when a hospital is not able to conduct the test on their own.”

In the present analysis, we use *Public Comfort with Sharing Health Data with Third-Party Commercial Companies for Patient Purposes* as Outcome 1, and *Public Comfort with Sharing Health Data with Third-Party Commercial Companies for Business Purposes* as Outcome 2. Demographic factors including self-reported health status, perceived healthcare access, and privacy concerns were our independent variables.

### Outcome 1: public comfort with sharing health data with third-party commercial companies for patient purposes.

To explore public comfort with sharing health data with third-party commercial companies for patient purposes, respondents answered questions about “how comfortable” they were with three statements regarding data sharing with third-party commercial companies, each along a 4-point Likert scale. Participants were asked “How comfortable are you with a third-party commercial company using your DNA and health information to improve the diagnosis and treatment of cancer in other patients?” and “How comfortable are you with a third-party commercial company developing predictions about how you will respond to a particular cancer treatment?: “not at all comfortable” (1), “somewhat comfortable” (2), “fairly comfortable” (3), and “very comfortable” (4). Participants were also asked “how true” it was that “The organizations that have my health information and share it can use large amounts of data to improve patient care”: “not true” (1), “somewhat true” (2), “fairly true” (3), and “very true” (4).

### Outcome 2: public comfort with sharing health data with third-party commercial companies for business purposes.

To examine participant comfort with sharing health data with third-party commercial companies for business purposes, participants were asked “How comfortable are you with a third-party commercial company storing your DNA and health information?”; “How comfortable are you with a third-party commercial company sharing predictions about how you will respond to cancer treatment with insurance companies?”; and “How comfortable are you with a third-party commercial company selling de-identified health information to a pharmaceutical company?”. “Business purpose” in this research is understood as storage of health data beyond the purposes of clinical care and sharing information with third-party commercial companies to improve their own business processes without explicitly stated direct benefit to patients. Respondents were provided with four options “not at all comfortable” (1), “somewhat comfortable” (2), “fairly comfortable” (3), and “very comfortable” (4). Indices for data use for patient purposes and business purposes were then calculated as the sum of participant responses to the three questions in each index divided by the number of questions. The Cronbach’s alpha for these questions was 0.766 for comfort with sharing health data with third-party commercial companies for patient purposes and 0.786 for comfort with sharing health data for business purposes, suggesting good internal consistency.

#### Demographic factors.

Demographic factors reported in this study include sex, age, race and ethnicity, education, income, and employment. The survey fielded by NORC provided with only two options for sex, male and female. Age was divided into four groups: 18–29, 30–44, 45–59, and 60+. Categories for race and ethnicity include “white, non-Hispanic”, “black, non-Hispanic”, “other, non-Hispanic”, “Hispanic”, “multiracial, non-Hispanic”, and “Asian, non-Hispanic”. Education was divided into four groups: less than high school, high school graduate, some college, or bachelor’s degree or above. Employment was grouped into four categories: employed, not-employed, retired, or not working due to disability or other reasons. Respondents were also asked to rate their own health (“Would you say that in general your health is… “poor” (1), “fair” (2), “good” (3), “very good” (4), “excellent” (5)).

#### Perceived healthcare access.

In addition to demographic information, we examine participant’s perceived ability to access healthcare services at a satisfactory level via the perceived healthcare access index. The index is based on various aspects of the healthcare experience and is evaluated here using a five-item index, asking “how true” (“not true”, “somewhat true”, “fairly true”, or “very true”) the following statements were for participants: (1) “The healthcare system in this country is easy to use”; (2) “I can get the healthcare I need when I need it”; (3) “I get all the information I need about my health from my healthcare provider”; (4) “I could access my electronic health record if I wanted to”; (5) “In general, I am satisfied with the treatment I receive from my healthcare provider”. The perceived healthcare access index was then calculated as the sum of participant responses to these five items and then divided by the number of questions. The Cronbach’s alpha was 0.820 for the perceived healthcare access index.

#### Privacy concerns.

To measure individual privacy concerns, respondent privacy attitudes were evaluated using a 4-item index, assessing their belief in the privacy protections of their healthcare system and whether they have concerns information about themselves is being misused or could be used in a way that is harmful to the respondent. The component questions for the privacy index are: “(1) My healthcare system respects my privacy; (2) I worry that private information about my health could be used against me; (3) I worry my health information is available to people who have no business seeing it; (4) There are some things I would not tell my healthcare providers because I can’t trust them with the information”. Each item asks respondents to rate “how true” each was for themselves on a Likert-type scale ranging from 1 (not true) to 4 (very true). The final privacy index score reflects the average of each participant’s response to these four questions. The first component question of the privacy index, “my healthcare system respects my privacy”, has been reversed-scored for inclusion in this index. The Cronbach’s alpha was 0.771 for the questions used in this index.

#### Data analysis.

Descriptive statistics were estimated on all variables and are used to describe the demographic characteristics, self-reported health status, perceived healthcare access, and privacy concerns of participants. A paired *t*-test examining the difference between comfort with sharing health data with commercial companies for patient purposes and comfort with sharing health data with commercial companies for business purposes was conducted to determine whether the difference between the two means is statistically significant.

Weighted ordinary least-squares (OLS) regression analysis was used to estimate the linear relationship between comfort with third-party commercial companies for patient and business purposes and each demographic and health variable separately. We then estimated a multivariable model with all demographic and health variables and conducted a stepwise regression model to identify a parsimonious set of variables that explained the greatest amount of variability in the two outcomes—comfort with sharing data with commercial companies for business or patient purposes. By using stepwise regression, we use a data-driven method for selecting variables in the final model. For the stepwise regression model, we set statistical significance at *α* = 0.05 (*p* < 0.002) for inclusion and *α* = 0.01 for exclusion, applying a Bonferroni correction to minimize Type I error. To enable comparison of effect sizes, regression coefficients were normalized (mean = 0, SD = 1).

## Results

### Sample demographics.

The resulting weighted sample of 1841 participants shows a near even split between male and female participants (49.05% male). Approximately 12% of participants were under the age of 29, and 31% of participants were over the age of 60. Nearly 60% of participants identified as white non-Hispanic, 15% as black, non-Hispanic, 19% as Hispanic, 3% as Asian, non-Hispanic, 2% of participants identified race and ethnicity as “other”, and 3% identified as multiethnic, results that are consistent with 2016 data from the US Census Bureau (12.3% of the US population identifies as black or African-American, non-Hispanic). Nearly half of participants completed some college (45.68%), and 33.13% of participants have a bachelor’s degree. While the proportion of participants with a bachelor’s degree is consistent with national percentages (30%, 2016 census data), the proportion of participants with some college, no degree is much higher in this study than national percentages (21%, 2016 census data). Just over half of participants (59%) made an income < $60,000, consistent with the median household income for 2018 (Guzman, 2019). Over half of participants (60%) had employment. Of the health questions included in this analysis, 89% of study participants reported having health insurance of some type, which is slightly lower than reported national percentages—92% of the US population according to the 2018 US Census (US Census Bureau, 2019). The mean self-reported health score of participants was 3.08, suggesting that on average, the respondents self-reported their own health as “good” (Table 1).

### Public comfort with sharing healthcare data with third-party commercial companies.

**Fig. 1:**
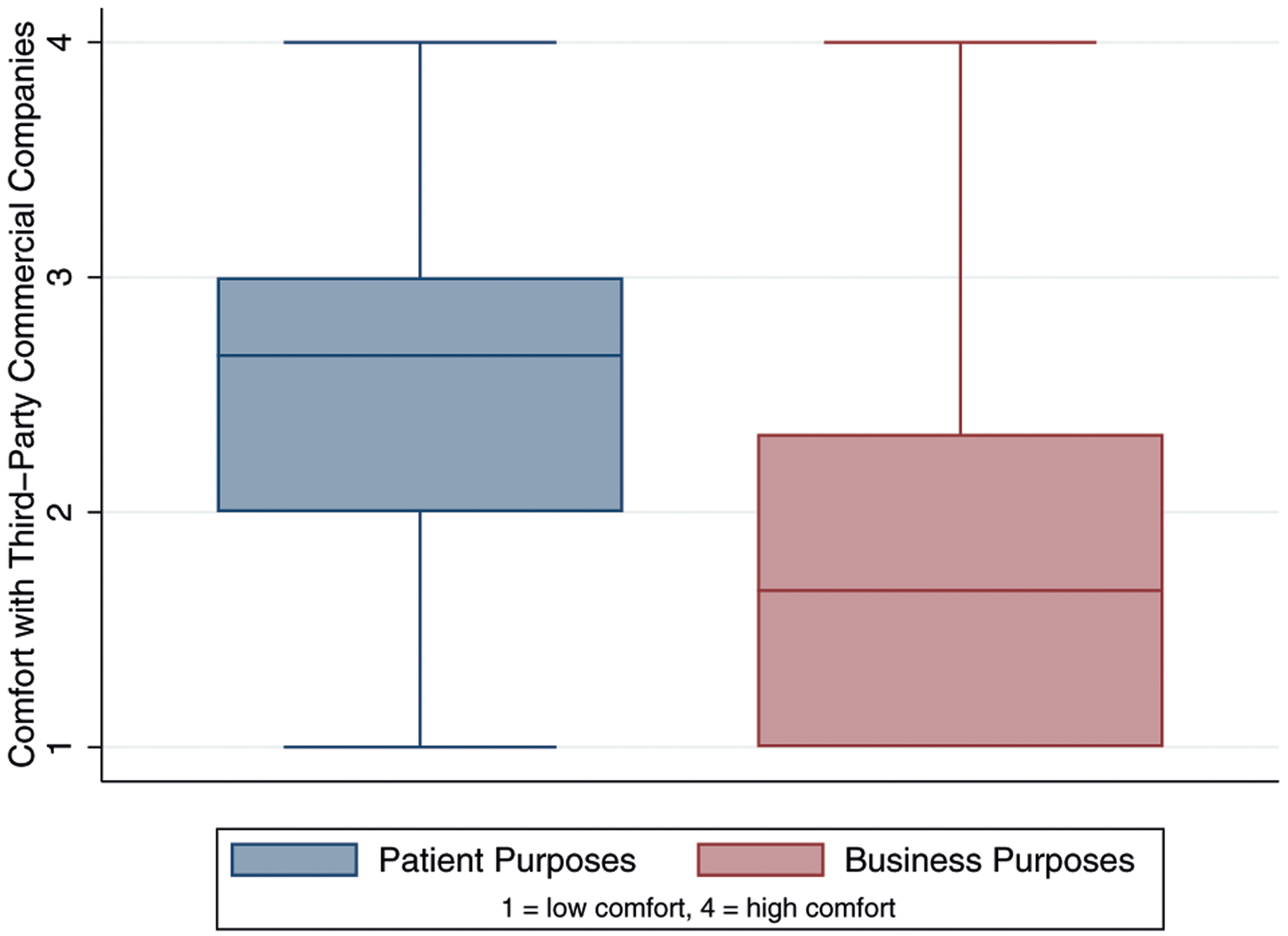
Comfort with third-party commercial companies. Description: Box plot distributions of comfort with sharing health data with third-party commercial companies for patient purposes and comfort with sharing health data with third-party commercial companies for business purposes. Low comfort is indicated by 1; high comfort is indicated by 4.

Public comfort with sharing health data with third-party commercial companies was evaluated using two three-item indices: (1) comfort with sharing health data with third-party commercial companies for patient purposes, and (2) comfort with sharing health data with third-party commercial companies for business purposes (Table 2). The resulting mean of comfort with sharing data with third-party commercial companies for patient purposes was 2.54 (SD = 0.81) or between “somewhat comfortable” and “fairly comfortable”. Roughly half of participants indicated that they were either fairly for very comfortable sharing data with third-party commercial companies for patient purposes (53.39% are comfortable with a third-party commercial company using their DNA and health information to improve the diagnosis and treatment of cancer in other patients, 49.16% are comfortable with third-party commercial companies developing predictions about how they will respond to a particular cancer treatment, and 47.80% believe that the organizations that have their health information and share it can use large amounts of data to improve patient care). Comfort with sharing health data with third-party commercial companies for business purposes had a resulting mean of 1.93 (SD = 0.85) or “somewhat comfortable”. One quarter to one-third of participants indicated they were either fairly or very comfortable with each of the component questions in comfort with sharing health data third-party commercial companies for business purposes (29.90% are comfortable with a third-party commercial company storing their DNA and health information, 31.02% are comfortable with a third-party commercial company sharing predictions about how they will respond to cancer treatment with insurance companies, and 24.39% are comfortable with a third-party commercial company selling de-identified health information to a pharmaceutical company). Figure 1 shows the distributions of the two indices.

A paired *t*-test was conducted on both comfort indices, the results of which show that there is a statistically significant difference between comfort with sharing health data with third-party commercial companies for patient purposes and comfort with sharing health data with third-party commercial companies for business purposes only, paired *t* = 39.84, *p* < 0.001.

### Perceived healthcare access.

The resulting mean index score for the perceived healthcare access index was 2.82 (SD = 0.75), which corresponds to “fairly true” for these questions, indicating fairly high confidence in participants’ ability to access healthcare services at a satisfactory level (Table 3). One-third (37.48%) of participants responded that it was fairly or very true that the healthcare system in the United States is easy to use, 70.23% responded that it was fairly or very true that they could get the healthcare they needed when they needed it, 62.42% responded that it was fairly or very true that they get all the information they needed about their health from their healthcare provider, 67.19% of participants responded that it was fairly or very true that they could access their electronic health record if they wanted to, and 73.01% of participants responded that it was either fairly or very true that they were satisfied with the treatment they received from their healthcare provider.

### Privacy concerns.

Participant attitudes toward privacy were assessed using a four-item index examining various facets of privacy in healthcare (Table 4). Just over half of participants (52.69%) responded that it was fairly or very true that their healthcare system respected their privacy, 35.58% responded that it was fairly or very true that they were worried health information could be used against them, 40.96% of participants indicated that it was fairly or very true that they worried their health information is being inappropriately accessed, and 24.12% responded that it was fairly or very true that they would withhold certain types of information from their care providers because of a lack of trust. One item in the index, “my healthcare system respects my privacy” was reversed so that higher Privacy Index scores consistently indicated greater privacy concerns. The resulting mean privacy attitudes index score was 2.22 (SD = 0.78), or a privacy confidence of “somewhat true”.

### Univariate model.

Examination of comfort with sharing health data with commercial companies for patient and business purposes by demographic variables and privacy attitudes display slight increases and statistically significant differences in comfort with sharing health data with commercial companies according to age, with participants between the ages of 45–59 indicating decreased comfort with sharing health data with third-party commercial companies for patient purposes compared to other age groups (b* = −0.102, *p* = 0.032). Education displayed a small trend, with comfort with sharing health data with third-party commercial companies for patient purposes increasing as education increased (possession of a bachelor’s degree: b* = −0.197, *p* = 0.002). Examination of privacy attitudes and comfort with sharing health data with third-party commercial companies reveals that as privacy concerns increase, comfort with sharing health data with third-party commercial companies for both patient (b* = −0.260, *p* = 1.9 × 10^−14^) and business purposes (b* = −0.264, *p* = 5.7 × 10^−14^) decreases (Table 5).

### Stepwise regression models.

Demographic predictors of comfort with sharing health data with third-party commercial companies *for patient purposes* show that in the multivariable model, 11% of the variability can be explained by demographic differences, perceived healthcare access, and attitudes towards privacy in the Bonferroni-corrected stepwise regression model. Five variables remained in the final regression model for comfort with sharing health data with third-party commercial companies for patient purposes: sex, age, education, perceived healthcare access, and privacy concerns. Possession of a bachelor’s degree was the strongest demographic predictor of increased comfort with sharing health data with third-party commercial companies for patient purposes (b* = 0.205, *p* = 0.0009). Examination of demographic predictors of public comfort with sharing health data with third-party commercial companies *for business purposes* show that in the multivariable model, 9.78% of variability can be explained by demographic differences, perceived healthcare access, and attitudes towards privacy. In the Bonferroni-corrected stepwise regression model, five variables remained in the final business purpose model: sex, education, employment, perceived healthcare access, and privacy concerns, which displayed the strongest association with comfort with sharing health data with third-party commercial companies for business purposes. As privacy concerns decreased, comfort with sharing health data with third-party commercial companies increased for both patient purposes (b* = −0.223, *p* = 6.9*10^−10^) and business purposes (b* = −0.246, *p* = 4.5*10^−12^) (Table 6).

## Discussion

To better understand the public’s comfort with sharing health data with third-party commercial companies, this study sought to examine differences in comfort with sharing health data for patient purposes and comfort with sharing health data for business purposes, and identify what demographic variables contributed to increased or decreased public comfort with sharing health data with third-party commercial companies. We also examined the relationship between privacy attitudes and comfort with sharing health data with commercial companies. Survey results revealed significantly less comfort with sharing health data with commercial companies for business purposes than patient purposes.

Demographic factors significant to comfort with sharing health data with third-party commercial companies for patient purposes include age and education. Employment is the only demographic variable significant to comfort with sharing health data with third-party commercial companies for business purposes. Notably, self-reported health status did not persist in the final model presented here, despite the significance of health status in other studies (Kim et al., 2017; Tikoo, 2014; Weitzman et al., 2010). One of the most significant findings of this study is that comfort with sharing health data with commercial companies for patient purposes *increased* with educational attainment, and that comfort with sharing health data with commercial companies for business purposes *decreased* with educational attainment. Although previous studies have identified an inverse relationship between willingness to share information and education, this study reveals that communicating the patient-centered motives for sharing health information with third-party commercial companies may reverse that trend. Perceived healthcare access was strongly associated with comfort with sharing health data with third-party commercial companies for patient purposes, likely indicating that ease of access, and the overall sense that one’s own needs are being adequately met, increases personal motivation to extend that care to others as well. It is also likely that individuals with high healthcare satisfaction are more empowered consumers of healthcare resources and feel a greater sense of agency and control over their health data and healthcare experience.

Decreased privacy concerns or decreased worry about how healthcare data is used was associated with increased comfort with sharing health data with third-party commercial companies and remained in the final stepwise regression model. In the privacy concerns index, we asked patients whether it was very or not true that the healthcare system respected [their] privacy. Interestingly, only half of participants indicated that it was either fairly or very true that the healthcare system respected [their] privacy. Patient privacy is of paramount importance in healthcare, with HIPAA representing one of the few examples of comprehensive privacy law worldwide. That half of participants feel their health systems respect their privacy indicates the gap between what we have been able to provide patients with regard to their privacy and what they require now amidst this rapidly developing computing and big data environment. One third of participants indicated their concern that private information about their health could be used against them, and less than half of participants indicated concern that their health information was available to people who had no business seeing it. Alleviation of these fears of abuse may decrease privacy concerns and increase comfort with sharing health data with third-party commercial companies.

### Implications for research.

Healthcare research is rapidly becoming dependent on the large data sets provided by ePHI. Data partnerships with companies like Google are increasingly being sought in order to expand the data processing and research capabilities of healthcare systems. At a minimum, this research indicates the importance of promoting the patient-centered benefits of these partnerships at not only their announcement, but at their inception. Although healthcare systems anticipate tremendous benefits to their patients in the creation of these partnerships, it should not be assumed that the public automatically perceives these partnerships to be beneficial. While improvements to business efficiency and processes may benefit patient care, our study indicates that these connections may not be clear and should be made more explicit. The large difference between comfort with sharing data with commercial companies for business versus patient purposes suggests a need for further interrogation of the different predictors of these variables and deeper examination of the meaning the public has ascribed to commercial companies.

### Implications for policy and practice.

In this research we found that increasing privacy concerns results in decreased public comfort with sharing health data with third-party commercial companies for both patient and business purposes. That the public feels so inadequately protected signals an urgent need to reassess the privacy laws and regulations of healthcare, and to take quick steps to differentiate the manner in which healthcare systems use PHI from the manner in which personal data is used in other industries. One possibility is to consider nationwide adoption of the Texas Medical Privacy Act, which is one of the broadest and most strict medical privacy laws in the United States. Under the Texas Medical Privacy Act, (1) any organization that assembles, collects, stores, or transmits PHI, or (2) comes into possession of PHI, is subject to HIPAA (Solove and Schwartz, 2019). Texas adds the additional prohibition of re-identifying de-identified data under any circumstance (Luna, 2011). As third-party partnerships proliferate, application of HIPAA to only covered entities requires reexamination.

### Limitations.

As with any cross-sectional survey, this study offers a snapshot of patient beliefs and preferences and limited due to the nature of survey questions. Other aspects of patient and public privacy concerns, perceived healthcare access, and health that may provide a more complete portrait of the public’s comfort with sharing health data with third-party commercial companies may not be captured here. Measures used to examine “patient purposes” and “business purposes” represent only a small selection of uses for patient health data and dividing purposes in this manner necessarily flattens patient purpose and business purposes into distinct buckets when the reality of patient data use is much more intertwined and complicated. The statistically significant differences between these purposes seen in this research indicate that patients may not be aware of the relationships between these two purposes of use. Additionally, a stepwise regression model is a conservative model that eliminates factors that might be important to understanding patient and public comfort with sharing health data with third-party commercial companies.

The circumstances of data sharing and the privacy context in which that sharing will occur will continue to evolve as laws, expectations, and experiences of healthcare data sharing change. Longitudinal studies that evaluate changes in comfort with sharing health data with third-party commercial companies would be superior, especially in light of changing media coverage of these partnerships. In subsequent research, we will examine a sampling of media events and their potential effect on comfort with sharing health data with third-party commercial companies.

## Conclusion

This study revealed that educational attainment is associated with increased comfort with sharing health data with third-party commercial companies for patient purposes and decreased comfort with sharing health data with third-party commercial companies for business purposes, and privacy concern is strongly associated with less comfort with sharing health data with third-party commercial companies for both patient and business purposes. This study also revealed differences in comfort with sharing health data with third-party commercial companies explicitly patient-centered purposes versus business purposes with no explicitly stated patient benefit. Healthcare systems embarking on new third-party data partnerships to expand their ability to process and analyze health data can benefit from early identification and communication of the patient-centered benefits that will result from their third-party commercial partnerships. Healthcare systems can do more to provide reassurances that healthcare privacy will be protected, for example: communicating data protection efforts to the public at the time a new third-party partnership is announced, proactive acknowledgment of privacy concerns as privacy breaches unfold, and frequent communication of what healthcare systems are doing to mitigate privacy risks. More research is needed on attitudinal dimensions related to privacy (trust, comfort with researchers, quality analysts, and commercial companies, protections, and notification of data access) to better understand comfort with sharing health data with third-party commercial companies for patient and business purposes.

## Figures and Tables

**Table 1:** Demographic descriptive statistics (*N* = 1841).

	*N*	Frequency (%)
*Sex*		
Male	903	49.05
Female	938	50.95
*Age*		
18–29	227	12.33
30–44	554	30.09
45–59	483	26.24
60+	577	31.34
*Race/ethnicity*		
White	1086	58.99
Black, NH	273	14.83
Other, NH	30	1.63
Hispanic	358	19.45
Multiracial, NH	47	2.55
Asian, NH	47	2.55
*Education*		
Less than High School	73	3.97
High School	317	17.22
Some college	841	45.68
BA or above	610	33.13
*Income*		
<$60,000	1082	58.77
$60,000 or greater	759	41.23
*Employment*		
Employed	1112	60.40
Not employed	87	4.73
Retired	373	20.26
Disabled/other	269	14.61
*Insured*		
Is insured	1638	88.97
Is not insured	203	11.03
*Self-reported health*		
Range: 1 (poor) to 5 (excellent)		Mean: 3.08 (SD = 0.92)

**Table 2:** Descriptive statistics for survey questions used in indices measuring comfort with sharing health data with third-party commercial companies for patient purposes and comfort with sharing health data with third-party commercial companies for business purposes (*N* = 1841).

	Frequency (% fairly or very comfortable/fairly or very true)	Mean (SD)
*Comfort with sharing health data with third-party commercial companies for patient purposes*		
How comfortable are you with a third-party commercial company using your DNA and health information to improve the diagnosis and treatment of cancer in other patients?	53.39	2.58 (1.05)
How comfortable are you with a third-party commercial company developing predictions about how you will respond to a particular cancer treatment?	49.16	2.48 (1.02)
The organizations that have my health information and share it can use large amounts of data to improve patient care	47.80	2.56 (0.86)
Comfort with sharing health data with third-party commercial companies for patient purposes index (Cronbach’s *α =* 0.769)	Median: 2.67	2.54 (0.81)
*Comfort with sharing health data with third-party commercial companies for business purposes*		
How comfortable are you with a third-party commercial company storing your DNA and health information?	28.90	1.98 (1.01)
How comfortable are you with a third-party commercial company sharing predictions about how you will respond to cancer treatment with insurance companies?	31.02	2.00 (1.04)
How comfortable are you with a third-party commercial company selling de-identified health information to a pharmaceutical company?	24.39	1.81 (1.01)
Comfort with sharing health data with third-party commercial companies for business purposes index (Cronbach’s *α =* 0.786)	Median: 1.67	1.93 (0.85)

Range of indices: 1 = not comfortable sharing health data with third-party commercial companies; 2 = somewhat comfortable sharing health data with third-party commercial companies; 3 = fairly comfortable sharing health data with third-party commercial companies; 4 = very comfortable sharing data with third-party commercial companies.

**Table 3:** Descriptive statistics for survey questions used in indices measuring perceived healthcare access (*N* = 1841).

	Frequency (% fairly or very true)	Mean (SD)
*Perceived healthcare access index*		
The healthcare system in this country is easy to use	37.48	2.22 (0.98)
I can get the healthcare I need when I need it	70.23	3.02 (0.95)
I get all the information I need about my health from my healthcare provider	62.42	2.82 (0.97)
I could access my electronic health record if I wanted to	67.19	2.99 (1.06)
In general, I am satisfied with the treatment I receive from my healthcare provider	73.01	3.04 (0.92)
Healthcare Access index (Cronbach’s *α =* 0.820)	Median: 2.8	2.82 (0.75)

Range: 1 = “not true”; 2 = “somewhat true”; 3 = “fairly true”; 4 = “very true”.

**Table 4:** Descriptive statistics for survey questions measuring privacy concerns (*N* = 1841).

	Frequency (% fairly or very true)	Mean (SD)
*Privacy index*^a^		
My healthcare system respects my privacy^b^	52.69	2.63 (0.91)
I worry that private information about my health could be used against me	35.58	2.22 (1.07)
I worry my health information is available to people who have no business seeing it	40.96	2.38 (1.05)
There are some things I would not tell my healthcare providers because I can’t trust them with the information	24.12	1.89 (1.00)
Privacy index (Cronbach’s *α =* 0.771)	Median: 2.25	2.22 (0.78)

a Range: 1 = “not true”; 2 = “somewhat true”; 3 = “fairly true”; 4 = “very true”.

b This question has been reversed-scored for inclusion in this index.

**Table 5:** Univariate associations of demographic factors, perceived healthcare access, and privacy concerns with comfort with sharing health data with third-party commercial companies for patient purposes and business purposes (*N* = 1841).

Demographics	Patient purposes			Business purposes		
	b*	*p*-value	*R*^*2*^	b*	*p*-value	*R*^*2*^
*Sex*						
Male	ref			ref		
Female	−0.037	0.25	0.001	−0.042	0.20	0.002
*Age*						
18–29	ref			ref		
30–44	−0.078	0.085	0.007	−0.035	0.49	0.005
45–59	−0.102	0.032		−0.091	0.087	
60+	−0.029	0.53		−0.027	0.61	
*Race/ethnicity*						
White	ref			ref		
Black, NH	−0.028	0.37	0.005	0.034	0.31	0.005
Other, NH	−0.029	0.38		−0.014	0.64	
Hispanic	−0.062	0.067		0.021	0.55	
Multiracial, NH	−0.031	0.29		−0.039	0.15	
Asian, NH	0.004	0.90		0.039	0.28	
*Education*						
Less than High School	ref			ref		
High School	0.098	0.14	0.014	0.001	0.99	0.011
Some college	0.126	0.034		−0.045	0.55	
BA or above	0.197	0.002		−0.117	0.14	
*Income*						
<$60,000	ref			ref		
$60,000 or greater	0.059	0.069	0.003	−0.028	0.41	0.001
*Employment*						
Employed	ref			ref		
Not employed	0.032	0.32	0.003	0.087	0.13	0.009
Retired	0.022	0.44		0.014	0.63	
Disabled/other	−0.037	0.29		−0.034	0.28	
*Insured*						
Has insurance	ref			ref		
Does not have insurance	−0.060	0.057	0.004	0.021	0.46	0.001
*Self-reported health*						
Poor	ref			ref		
Fair	0.013	0.84	0.012	0.010	0.88	0.003
Good	0.043	0.57		0.062	0.38	
Very good	0.073	0.29		0.010	0.88	
Excellent	0.119	0.021		0.022	0.67	
*Perceived healthcare access index*						
Perceived healthcare access	0.204	6.0*10^−10^	0.041	0.154	3.8*10^−06^	0.024
*Privacy concerns*						
Privacy concerns	−0.260	1.9*10^−14^	0.068	−0.264	5.7*10^−14^	0.070

b* = standardized beta.

**Table 6:** Stepwise regression modeling of predictors of comfort with sharing health data with third-party commercial companies for patient purposes and comfort with sharing health data with third-party commercial companies for business purposes (*N* = 1841).

	Patient purposes multivariable stepwise Bonferroni corrected (α = 0.002)		Business purposes multivariable stepwise Bonferroni corrected (α = 0.002)	
	Model *R*^*2*^	0.1117	Model *R*^*2*^	0.0978
	b*	*p*-value	b*	*p*-value
*Sex*				
Male	ref		ref	
Female	−0.056	0.062	−0.064	0.037
*Age*				
18–29	ref			
30–44	−0.104	0.02		
45–59	−0.154	0.0012		
60+	−0.117	0.012		
*Education*				
Less than High School	ref		ref	
High School	0.089	0.16	−0.040	0.62
Some college	0.133	0.021	−0.069	0.37
BA or above	0.205	9.0*10^−4^	−0.145	0.079
*Employment*				
Employed			ref	
Not employed			0.071	0.034
Retired			−0.037	0.22
Disabled/other			−0.060	0.053
*Perceived healthcare access index*				
Perceived healthcare access	0.140	5.4*10^−5^	0.070	0.051
Privacy concerns index Privacy concerns	−0.223	6.9*10^−10^	−0.246	4.5*10^−12^

b* = standardized beta.
